# Comparing orthopaedic paediatric trauma in a rural district general hospital and an urban major trauma centre: A retrospective cohort study

**DOI:** 10.1016/j.amsu.2021.102276

**Published:** 2021-04-13

**Authors:** Mrs J. Dunn, Mr M. Amer, Ms M. Soupashi

**Affiliations:** a Trauma and Orthopaedics Department, Ninewells Hospital, James Arrott Drive, Dundee, Scotland, DD2 1SG, United Kingdom; b Trauma and Orthopaedics Department, Raigmore Hospital, Old Perth Road, Inverness, Scotland, IV2 3UJ, United Kingdom

**Keywords:** Trauma, Rural, Paediatric, Urban, Cohort

## Abstract

**Background:**

Studies from countries such as Australia and South Africa have demonstrated a difference in the types of injury managed in rural hospitals compared to larger, urban hospitals and so conclude staff require a different skill-set to work in these environments. There is some evidence this attitude may be prevalent amongst UK surgical trainees, resulting in difficulty recruiting to rural settings. In addition, studies have compared mortality in paediatric trauma patients in rural and urban hospitals, but none have described types of injury or orthopaedic operations required.

We hypothesise the distribution of operative, orthopaedic paediatric trauma in a rural district general and an urban major trauma centre will not differ significantly in terms of patterns and mechanism of injury, orthopaedic intervention or time to theatre.

**Materials/methods:**

All operative paediatric patients (0–15yrs) seen during an acute orthopaedic take at a rural district general and an urban major trauma centre were included. Non-operative admissions were excluded. Patients were identified using daily trauma work lists from each site. Outcomes were age, injury type, operation, time to theatre, seasonality and mechanism.

**Results:**

183 patients from the urban hospital and 103 from the rural were identified. There were no significant differences found in age of patient, seasonality or time to theatre between cohorts (p > 0.05). There were also broadly similar patterns of injury and operations performed in both groups, although k-wiring was more often employed in the rural cohort than the urban (27% vs 17% of total operations). There were more bicycle and shinty related injuries in the rural cohort, and equine related in the urban.

**Conclusions:**

Paediatric trauma admissions do not vary significantly between rural and urban trauma centres, although the types of procedure performed may be less conservative in a rural hospital. This may be due to geography or differences in ED practice.

## Highlights

• Distribution of operative, orthopaedic paediatric trauma in a district general compared with a major trauma centre.
• Rural hospital pediatric orthopaedic trauma admission compared with an urban hospital.
• Retrospective cohort study.
• Types of injury and operation performed were compared.
• Mechanism and time to theatre were compared.

## 1 Hypothesis

Quantative and qualitative studies have concluded that patient presentation in rural and urban hospitals differ in terms of trauma workload, procedural skills required by staff and variety of cases seen [1,2].

Many of these studies have been undertaken in an overseas context that differs significantly from rural hospital work in the UK, and may falsely lead UK surgical trainees or consultants to believe they require additional training to work in a rural hospital in the UK (often district generals) compared to an urban major trauma centre, making this a less attractive career option. We hypothesise that the distribution of operative paediatric trauma in a rural district general and an urban major trauma centre in Scotland will not differ significantly in terms of patterns of injury, mechanism of injury, orthopaedic intervention and time to theatre.

## 2 Aim

Our aim was to compare patterns of rural and urban operative paediatric trauma, to evaluate potential trends and identify areas for service improvement or any difference in expertise required by the surgical staff working in those areas. Many studies have examined differences in mortality between rural and urban centres, but none have described types of injury sustained or what orthopaedic operations were required in any detail in a paediatric population.

## 3 Introduction

Studies comparing rural and urban paediatric trauma generally show a higher mortality associated with trauma in a rural setting [[3], [4], [5], [6]]. A 2018 American study [7] found that rates of non-accidental paediatric trauma were similar in a rural and urban setting, and that mortality for non-accidental trauma increased in both of these settings compared with accidental injuries.

A very large 2017 American study (50,000 patients) found no significant difference between mortality in rural vs urban adult trauma, although patients who did die tended to within the first 24 h in rural settings, rather than later in urban major trauma centres [8]. Many other papers [[9], [10], [11], [12], [13]] have reported higher mortality rates for all (adult and paediatric) trauma in rural centres vs urban hospitals, although the major trauma centre status of many of these institutions in cities may account for this.

No studies, however, were found which compared the type of trauma, time to theatre, mechanism or specific orthopaedic operations performed in this patient group.

## 4 Methods

### 4.1 Study design

This is a retrospective cohort study comparing two centres-a rural, district general hospital in the Scottish highlands and a major trauma centre teaching hospital situated in a large Scottish city.

### 4.2 Inclusion/exclusion criteria

All operative patients aged 0–15 (up to the day before their 16th Birthday) who presented to a rural district general hospital in the Highlands of Scotland, or to an urban Major Trauma Centre in a Scottish city, between April 1st 2019- April 1st 2020 were included in the study. Additionally, they must have been seen on an acute orthopaedic take. Patients were identified using the Orthopaedic daily trauma work lists from both centres.

Patient were excluded if they did not have the proposed operation on the trauma worklist, or if they underwent an intervention in the Emergency Department rather than the operating theatre (for example closed manipulation of a fracture). Patients who had an acute presentation to orthopaedic teams but did not undergo operative intervention were also excluded.

The ‘SPSS’ program- Statistical Package for the Social Sciences-was used for statistical analysis of data. Ethical approval was not required. This work has been reported in line with the Strengthening the Reporting of Cohort Studies in Surgery, or ‘STROCSS’, criteria [14]. This work is registered at ‘Clinical Trials. Gov’. The research identifier is NCT04760886 [15].

## 5 Results

183 patients from the urban centre were identified and 103 patients from the rural hospital. Follow up was 1 year for each cohort.

### 5.1 Comparison of results

**Table:** 

	Rural	Urban	Significance
**Age (median)**	8.3	8.4	p > 0.05
**Presentation over winter months (patients)**	19 (18%)	20 (11%)	p > 0.05
**Presentation over summer months (patients)**	32 (30%)	73 (39%)	p > 0.05
**Average time to theatre (hrs)**	16.1	16.2	p > 0.05
**Operation day of admission (patients)**	10 (10%)	24 (13%)	p > 0.05
**Operation outwith 24 h of admission (patients)**	1 (1%)	8 (4%)	p > 0.05

We found that patient demographics such as age did not differ significantly between groups, nor interestingly did time to theatre. There seemed to be a greater number of patients admitted over winter months in the rural hospital (18%) vs the urban hospital (11%), but again this result was not statistically significant. There were similar proportions of patients having more urgent operations (the same day they were admitted), and likewise a similarly small number waiting longer than 24 h for their operations.

**Figure:**
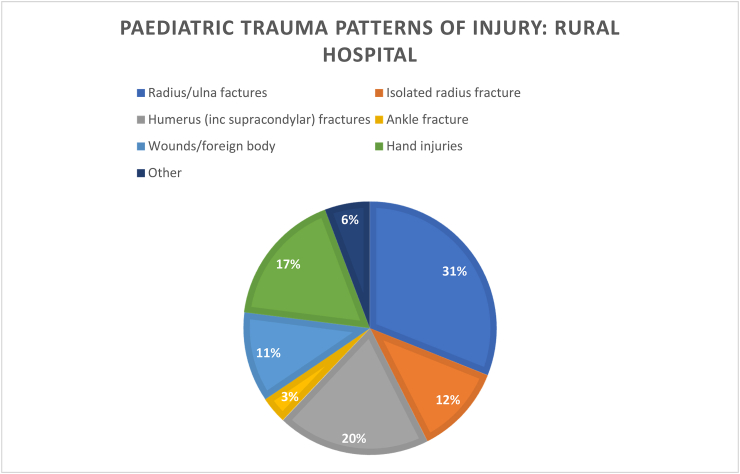

**Figure:**
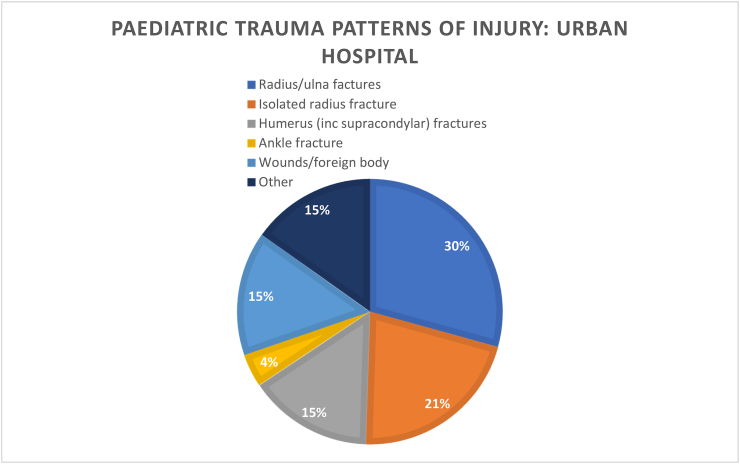

Injury patterns in both rural and urban centres were seen to be similar, although orthopaedic services received hand trauma referrals in the rural centre but did not in the urban centre. P=>0.05.

**Figure:**
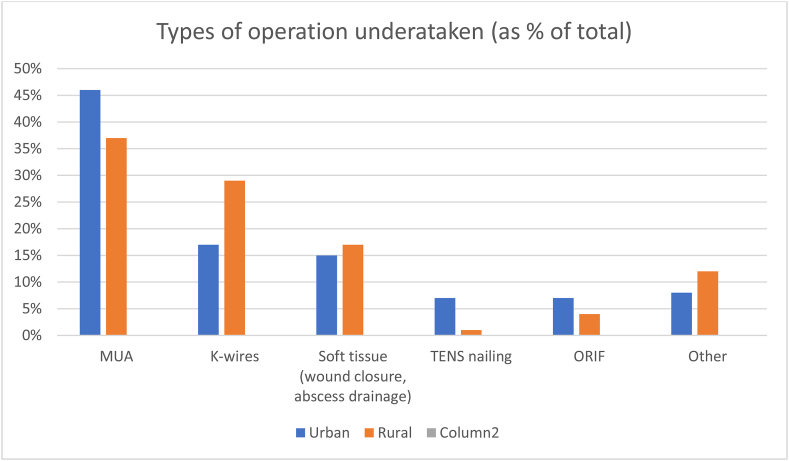

### 5.2 Mechanism of injury

We found numbers of injuries caused by trampolines, monkeybars and playing football were similar in both cohorts. Whilst there was only 1 patient in the urban area who sustained injures from a bicycle, there were 9 in the rural cohort. There were however 8 pony/horse related injuries in the urban cohort, but none in the rural. There were 2 shinty-related injuries in the rural cohort.

## 6 Discussion

This study was undertaken to address the perceived limitations sometimes expressed by surgical trainees and consultants of working in a rural, district general setting rather than a major trauma centre. Recruitment has been challenging to rural district general hospitals in Scotland in particular, as highlighted by the Royal College of Surgeons of Edinburgh's 2016 paper on the delivery of rural surgical care where they state: ‘provision of elective and emergency surgical services to small communities spread over a considerable geographical area becomes more problematic’, with reference to training needs of surgeons employed in these contexts [16].

As stated in the introduction, there is a deficit in the number of papers examining the types of injury seen in an urban Major Trauma Centre and a rural or district general hospital, or the orthopaedic interventions undertaken. A Pubmed search using the search terms ‘major trauma centre district general’ yielded no results comparing patterns of injury or orthopaedic interventions. One 2019 UK paper was found which documented patterns of open, complex paediatric trauma injury and compared them to patterns of injury in the Elderly in an English major trauma centre [17]. There were 33 complex, open injuries documented that year in their paediatric population, compared with no such injuries in the Scottish MTC discussed in this paper. This shows that individual MTC's can have a very different mix of trauma presentations, a factor which may somewhat limit the broad application of this study.

A further 2020 study from the UK has demonstrated a 50% reduction in all orthopaedic trauma referrals to the UK MTC hospital during the COVID-19 pandemic [18]. This study looks at trauma take for two centres from 1st April 2019–2020. This would incorporate the first month of what is now recognised as the start of the UK national lockdown (1^ST^ of March 2020), so numbers in this study may have been larger had this month not been included.

A limitation of this study is the definition of a rural district general hospital. Whilst the hospital in the Scottish Highlands discussed in this paper sees a considerable number of patients who live in a rural area compared to the bigger Major Trauma Centre, the geography of Scotland means than even patients presenting to a MTC in a city may live in surrounding ‘rural’ villages. Likewise, the Highland hospital we class as ‘rural’ is surrounded by a much more dense population than even more isolated hospitals, such as those in the Scottish Western Isles or Shetland isles. Further work comparing those centres would be illuminating.

Other limitations include the retrospective nature of our analysis- an ongoing, prospective audit of trauma cases or a national database would be of use in analysing the differing trauma trends in various hospitals across Scotland, and in informing the training needs of doctors working in these fields. Although we have not demonstrated any significant difference in case variety in our study, the same may not be true of other orthopaedic areas such as adult trauma, or other surgical specialties.

## 7 Conclusions

This study would suggest that, between a rural and urban hospital, age of patient, season of presentation and time to theatre does not differ significantly in a paediatric trauma population. It would also suggest that the types and distribution of trauma do not differ significantly, suggesting an orthopaedic surgeon does not need a ‘rural’ skillset to work in such a setting. Nor does a rural hospital have a more limited range of presentations. The types of operations performed were broadly similar, however more k-wires were employed in a rural setting, rather than manipulation under anaesthetic only.

Mechanism of injury analysis showed that monkeybars, trampolines and football remained the most common cause of injury in both groups in equal proportions, although there were more equine related injuries in the urban population, and more bicycle and shinty related injuries in the rural cohort.

Future research could focus on the smaller, even more remote rural district general hospitals in Scotland to establish if a significant difference in trauma variety exists in these contexts compared with a major trauma centre. Adult trauma could be included or other surgical presentations outwith trauma and orthopaedics.

## Ethical approval

No ethics approval required, discussed with local Caldicott guardian.

## Source of funding

None.

## Author contribution

Ms J Dunn-study design, coordination, data collection for one hospital, statistical analysis, write up.

Mr M Amer-study design, data collection for one hospital, re-checking data collection, assisted in making coloured tables, proof-reading write up.

Ms Soupashi-data collection for other hospital (all), assisted in write-up of results section.

## Trial registry number

1 Name of the registry: Clinical Trials. gov.

2 Unique Identifying number or registration ID: NCT04760886.

3 Hyperlink to your specific registration (must be publicly accessible and will be checked): https://clinicaltrials.gov/ct2/show/NCT04760886.

## Guarantor

Mrs J M Dunn (corresponding author).

## Consent

–

## Provenance and peer review

Not commissioned, externally peer-reviewed.

## Declaration of competing interest

None.
